# From Multifunctionality to Multiple Ecosystem Services? A Conceptual Framework for Multifunctionality in Green Infrastructure Planning for Urban Areas

**DOI:** 10.1007/s13280-014-0510-2

**Published:** 2014-04-17

**Authors:** Rieke Hansen, Stephan Pauleit

**Affiliations:** Technische Universität München, Emil-Ramann-Straße 6, 85354 Freising, Germany

**Keywords:** Social–ecological systems, Ecosystem services, Green Infrastructure, Urban planning, Environmental planning

## Abstract

Green infrastructure (GI) and ecosystem services (ES) are promoted as concepts that have potential to improve environmental planning in urban areas based on a more holistic understanding of the complex interrelations and dynamics of social–ecological systems. However, the scientific discourses around both concepts still lack application-oriented frameworks that consider such a holistic perspective and are suitable to mainstream GI and ES in planning practice. This literature review explores how multifunctionality as one important principle of GI planning can be operationalized by approaches developed and tested in ES research. Specifically, approaches developed in ES research can help to assess the integrity of GI networks, balance ES supply and demand, and consider trade-offs. A conceptual framework for the assessment of multifunctionality from a social–ecological perspective is proposed that can inform the design of planning processes and support stronger exchange between GI and ES research.

**Electronic supplementary material:**

The online version of this article (doi:10.1007/s13280-014-0510-2) contains supplementary material, which is available to authorized users.

## Introduction

Within the last few years green infrastructure (GI) has become a popular concept to guide planning toward sustainable land use (Ahern 2007; Mazza et al. 2011). Within Europe, for instance, the European Union’s environmental policy promotes GI as a planning approach applicable at different spatial levels (ibid.). Recently, the European Commission launched a strategy titled “Green Infrastructure—Enhancing Europe’s Natural Capital,” which aims at mainstreaming GI in spatial planning and territorial development in order to consciously consider the manifold benefits humans obtain from nature. GI is defined as a “strategically planned network of natural and semi-natural areas with other environmental features designed and managed to deliver a wide range of ecosystem services” (European Commission 2013). In contrast to monofunctionally planned “gray” infrastructure, GI enhances and synergizes benefits provided by nature.

Despite its increasing popularity, GI remains a broad and elusive concept. One reason for this is its broadness of scale: The term can be used for regional or national ecological networks (e.g., Weber and Allen 2010), green space networks for urban areas (e.g., Kambites and Owen 2006), as well as local storm water management projects (e.g., Ahern 2010). In the scientific literature, GI planning is discussed as based on various principles or guidelines such as multifunctionality, connectivity, or collaborative planning (Table 1). However, the specific sets of principles which characterize GI planning vary (e.g., Benedict and McMahon 2006; Kambites and Owen 2006; Pauleit et al. 2011). Overlaps with other concepts that share principles such as connectivity or strategic and adaptive planning (e.g., Ahern 1995) further complicate the discussion on GI as a distinctive approach. Accordingly, GI planning represents more of a synthesis of different planning approaches than a completely new approach (Mell 2009). Rather, the defining characteristic of GI planning is that it is a melting pot for innovative planning approaches in the field of nature conservation and green space planning.

**Table 1:** Green infrastructure planning principles

Green infrastructure planning principles (based on Benedict and McMahon 2006; Kambites and Owen 2006; Pauleit et al. 2011)
Approaches addressing the green structure
Integration: Green infrastructure planning considers urban green as a kind of infrastructure and seeks the integration and coordination of urban green with other urban infrastructures in terms of physical and functional relations (e.g., built-up structure, transport infrastructure, and water management system)
Multifunctionality: Green infrastructure planning considers and seeks to combine ecological, social and economic/abiotic, biotic and cultural functions of green spaces
Connectivity: Green infrastructure planning includes physical and functional connections between green spaces at different scales and from different perspectives
Multi-scale approach: Green infrastructure planning can be used for initiatives at different scales, from individual parcels to community, regional, and state. Green infrastructure should function at multiple scales in concert
Multi-object approach: Green infrastructure planning includes all kinds of (urban) green and blue space; e.g., natural and semi-natural areas, water bodies, public and private green space like parks and gardens
Approaches addressing governance process
Strategic approach: Green infrastructure planning aims for long-term benefits but remains flexible for changes over time
Social inclusion: Green infrastructure planning stands for communicative and socially inclusive planning and management
Transdisciplinarity: Green infrastructure planning is based on knowledge from different disciplines such as landscape ecology, urban and regional planning, and landscape architecture; and developed in partnership with different local authorities and stakeholders

Furthermore, the potential of GI planning to combine ecological and social perspectives is broadly acknowledged (Mell 2009). Due to its holistic approach, GI planning is considered to be more effective and able to handle more complexity than traditional planning for nature conservation or open space (Kambites and Owen 2006). In this light, GI planning appears to be especially suited for urban areas because these areas are characterized by the strong, dynamic interplay of ecological and social systems (e.g., Alberti et al. 2003; Pickett et al. 2011).

Examples of GI planning can be found especially in the US and in the UK, where GI was taken up and promoted by policy (Benedict and McMahon 2002; Kambites and Owen 2006). For other regions, such as Asia or Africa, scattered publications refer to the GI concept (e.g., Chang et al. 2012; Schäffler and Swilling 2013). Yet, often it remains to be clarified if planning practice actively adopted the concept or if it was only introduced by the authors on a theoretical level. In Europe, numerous initiatives to establish ecological networks exhibit overlaps with GI planning but rarely consciously relate to the concept (Mazza et al. 2011). Boosted by the EU-GI-strategy awareness of the concept will most likely further rise and questions on how to apply GI as a planning approach will become more important.

Apart from a few analytical studies of GI planning in practice (Sandström 2002; Lafortezza et al. 2013) and the presentation of some best practice examples (e.g., Mazza et al. 2011; Pauleit et al. 2011), research on how GI as a social–ecological approach can be applied is scant. In contrast to the frequent references to the concept, which recently can be found in scientific publications, little development of its theoretical foundation can be observed since its seminal description by Benedict and McMahon (2002, 2006) and the proposal of a conceptual framework to link ecological and social aspects by Tzoulas et al. (2007).

Consequently, GI research appears fragmented and lacks a distinctive theoretical foundation (Mell 2009). The lack of a specific theory of its own may be explained by the fact that GI principles such as ecological connectivity were adopted from landscape ecology (e.g., Ahern 2007; Chang et al. 2012). The concept of ES is also frequently adopted in GI literature to replace GI functions (e.g., Mazza et al. 2011; Lovell and Taylor 2013) but approaches for the operationalization of multifunctionality as a planning principle are still missing.

Developing a conceptual framework for multifunctionality could build an important foundation of GI theory and inform practitioners on crucial aspects in the design of planning processes from a social–ecological perspective. It would thus support mainstreaming GI in planning practice as pursued by European environmental policy. The synthesis of GI and ES theory into one framework seems promising, as ES research discusses several relevant aspects for multifunctional planning such as how to enhance ES in a beneficial way while avoiding trade-offs (e.g., Chan et al. 2012; Haase et al. 2012). Moreover, ES research helps to shed light on the interrelations between social and ecological systems and the integration of stakeholder perspectives in assessments (e.g., Diaz et al. 2011; Ernstson 2013).

Therefore, this paper aims at exploring possible linkages between GI and ES research with regard to multifunctionality. The review of GI and ES theory is an initial step to relate both fields of research and in so doing provide the ground for identifying opportunities for joint research with a view to support GI planning and implementation. The following is based on a review of GI and ES literature. The focus lies on studies for urban areas, but promising approaches or axiomatic theories from non-urban literature were not generally excluded. Using the Web of Knowledge, the search term “green infrastructure” was linked to “planning”; “framework”; or “strategy.” Furthermore, chapters of landmark environmental planning and urban ecology textbooks as well as policy guidance reports dealing with GI were included. Due to the extensive body of ES literature (Seppelt et al. 2011; Haase et al. 2014) the focus lies on publications that discuss the theoretical foundation of ES (e.g., relations between services and functions); that suggest the application of ES in planning processes; or that explore a social–ecological approach (e.g., frameworks for inclusion of stakeholder perspectives in ES assessments). Using a snowball approach, literature referenced in the reviewed papers was added. Overall, about 200 papers were reviewed (Electronic Supplementary Material, Appendix S1).

The GI and ES literature was reviewed for theoretical components that can be related to the concept of multifunctionality. Using GI theory as point of departure, ES theory was surveyed for complementary or additional aspects. Inspired by frameworks from ES and GI literature, components were then linked in an iterative process to form a conceptual framework for the assessment of multifunctionality.

## Foundations for Considering Multifunctionality

Before presenting the conceptual framework, this section defines basic terms used in the proposed framework such as functions and services because they are used differently in GI and in ES literature. Furthermore, we review the spatial levels on which GI can be considered and general frameworks for GI to illustrate the foundation for a framework of multifunctionality.

### From Functions to Services

#### Definition of Multifunctionality

Multifunctionality represents the holistic thrust of GI and can be—together with connectivity—considered as a core element of GI planning (Kambites and Owen 2006; Pauleit et al. 2011). The concept of multifunctionality in GI planning means that multiple ecological, social, and also economic functions shall be explicitly considered instead of being a product of chance (ibid.). Multifunctionality aims at intertwining or combining different functions and thus using limited space more effectively (Ahern 2011). The multiple functions should offer benefits for humans, for instance, in relation to human health or social cohesion, and likewise secure intact ecological systems (Tzoulas et al. 2007; Lafortezza et al. 2013).

#### Functions of GI

In literature on GI, its functions are usually listed without their further definition. They are, for example, grouped as ecological, social, and economic functions (Pauleit et al. 2011) or, following an alternative classification, as abiotic, biotic, and cultural functions of green spaces (Ahern 2007). These approaches usually capture a broad understanding of functions—ranging from soil development processes, support of species movement to physical recreation (e.g., Ahern 2007; Llausas and Roe 2012). Occasionally, ES classifications are transferred to GI approaches to replace functions (e.g., Mazza et al. 2011; Lovell and Taylor 2013). The latter causes a conceptual problem because in ES research functions and services are not considered as interchangeable.

#### Functions, Services, and Benefits in Research on ES

**Fig. 1:**
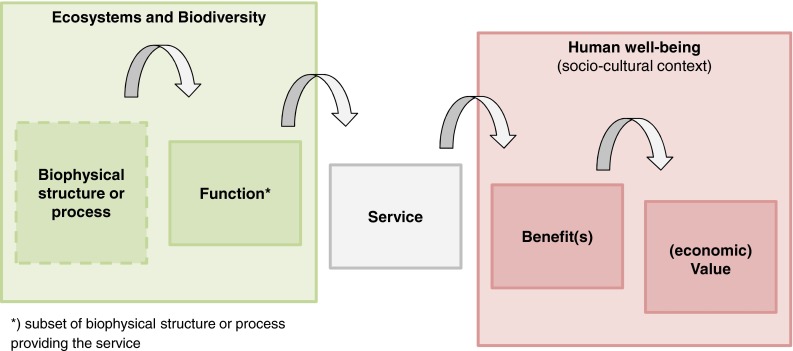
Cascade model for linking ecosystems to human well-being (adapted from Haines-Young and Potschin 2010 and de Groot et al. 2010)

The distinction between functions and services in ES research may help to achieve a more profound and differentiated understanding of functions and services. This distinction is important because the processes or functions of ecosystems such as soil formation may be crucial for their existence but not necessarily directly utilized by humans while a service per definition requires human beneficiaries (Fisher et al. 2009). Therefore, functions are discussed, for example, as “intermediate products” of ES (Boyd and Banzhaf 2007). This distinction is elaborated in the so-called ES cascade model by Haines-Young and Potschin (2010) (see Fig. 1). In this model, biophysical structures or processes (e.g., wetlands or net primary productivity) are the base for functions (e.g., slow passage of water). The functions can be the origin of services for humans (e.g., flood protection). These services lead to human benefit and valuation of those services (e.g., willingness to pay for wetland protection).

The cascade model could be applied to GI planning in order to better differentiate functions and services in GI approaches where functions are currently used in a fuzzy way, often meaning the same as services. Adopting a consistent use of terms and a clear distinction between functions and services would ensure that double counting due to overlaps between particular functions and services can be more easily detected (Hein et al. 2006). To avoid mixing GI functions and the concept of ES in the following sections, whenever feasible, the term “services” is used while “functions” refer to the ecological functioning of GI elements.

### Spatial Levels in GI Planning

**Fig. 2:**
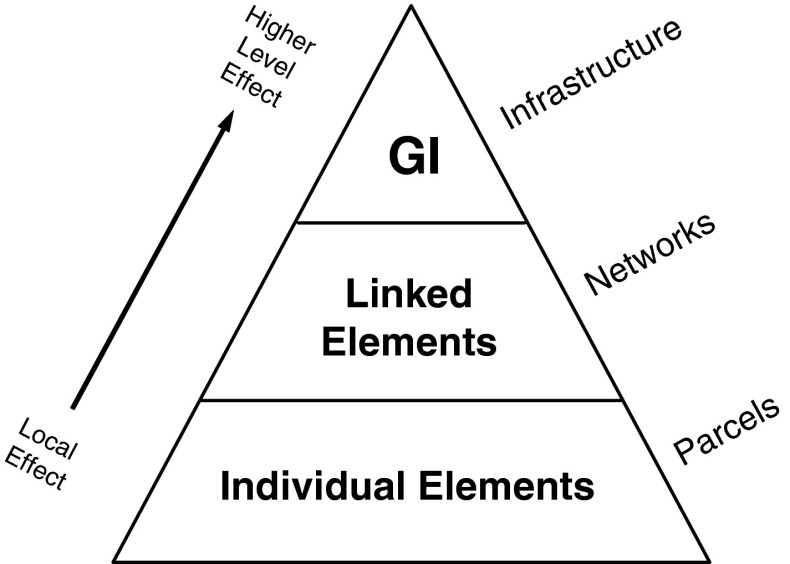
Multifunctionality can be assessed at different spatial levels (reproduced from Davies et al. 2006 with kind permission by the authors)

Three spatial levels that should be considered in GI planning are suggested by Davies et al. (2006) (see Fig. 2). Individual elements such as parks or rivers are the basis of GI. Site-specific assessments of multifunctionality can be applied for these single GI elements (Pauleit et al. 2011).

On the next spatial level different GI elements and the linkages between them are represented. They form a network that enables movement of species and flows of matter. These networks can be considered for areas of different sizes (e.g., neighborhood or city). At the highest level is GI, which is composed of interlinked networks of GI elements on the regional level. On these higher levels, multifunctionality can be used to assess this interrelated system of different types of green and open space that in its entirety provides multiple benefits (Ahern 2007).

The framework for the assessment of multifunctionality proposed in this paper makes no distinction between this highest level and the network level because, especially in urban areas, it is difficult to determine where the boundary between a network and (regional) GI lies. Thus in the following, GI elements are considered on one hand and on the other are networks as systems of individual elements and links between them in a defined area. These areas can range from a neighborhood to an entire urban region.

### Existing Frameworks for GI Planning

A couple of existing theoretical frameworks for GI planning offer a starting point to discuss which conceptual components should be integrated in GI planning. Tzoulas et al. (2007) propose a framework for GI in urban areas that provides the ground for linking ecological concepts such as ecosystem health to social concepts such as individual or community health. On this basis, Lafortezza et al. (2013) describe a framework for GI planning with five interlinked conceptual components: ES, biodiversity, social and territorial cohesion, sustainable development, and human well-being. The components of these frameworks, while illustrative, require further operationalization with methods that allow their systematic assessment and valuation in planning.

In contrast, practice-oriented outlines of GI planning can be found in publications of GI initiatives from the UK and US (e.g., Benedict and McMahon 2006; Davies et al. 2006; The North West Green Infrastructure Think Tank 2008). For instance, the “Five Steps to Green Infrastructure Planning” from The Mersey Forest (2011) consists of (1) partnerships and priorities; (2) data audit and resource mapping; (3) functional assessment; (4) needs assessment; and (5) intervention plan. These planning frameworks are usually more focused on the structuring of planning processes and inspired by case studies rather than on theoretical foundations.

A combination of elements from theoretical frameworks and planning process guidance can contribute to the scientific discourse on GI as well as inform practitioners on planning process design. This dual purpose is the aim of the proposed conceptual framework for multifunctionality.

## A Tentative Conceptual Framework for Assessing Multifunctionality in GI Planning

In the following, an attempt is made to outline a framework for assessing multifunctionality in GI planning that is linked to ES theory. The framework shall combine the current knowledge on GI and ES assessment and inform plan-making on how to determine options to conserve, strengthen, or enhance multifunctionality of urban green space. After introducing the structure of the framework the different dimensions are explained.

### Structure of the Framework

The overall frame for the assessment of multifunctionality is based on concepts for ES with a social–ecological perspective by Bastian et al. (2012), Diaz et al. (2011), and Ernstson (2013) as well as de Groot et al. (2010). The framework is structured in four dimensions: to determine the status quo in the analysis of the system, and the ecological and the social perspective are surveyed separately (dimension I and II). The ecological perspective aims at data collection on the capacity of the existing GI network to provide services. The social perspective covers the demand side. In valuation (dimension III), both perspectives are integrated and used to determine priorities for strategies and actions (dimension IV).

**Fig. 3:**
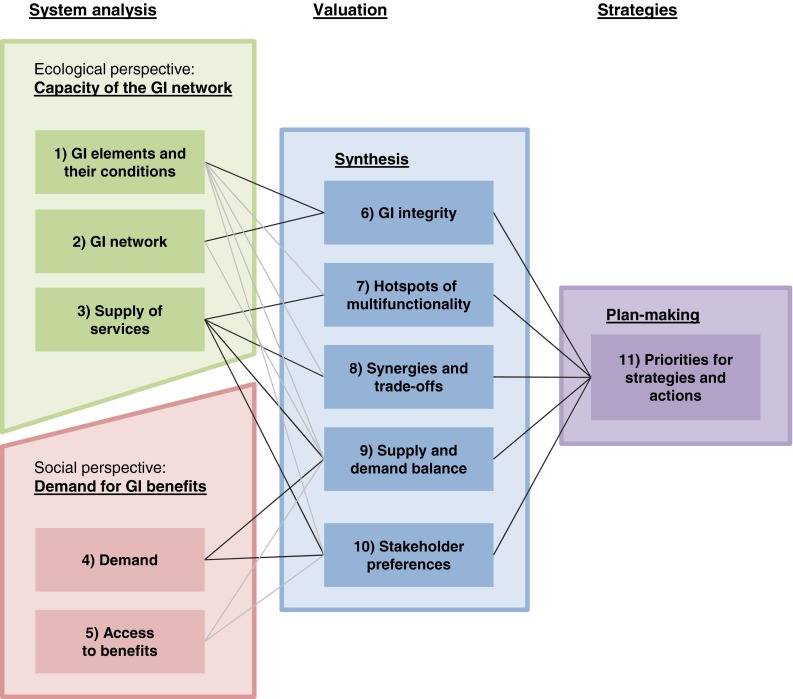
Conceptual framework for assessment of GI multifunctionality. The *boxes with numbers* represent different conceptual components derived from GI and ES literature. The *lines* map data flow from *left to right*. *Lines in black* indicate main relations between components while *gray lines* illustrate supporting relations

The different dimensions are filled with conceptual components from GI and ES research that can support a comprehensive determination of multifunctionality. Each component of the framework is represented by a number in Fig. 3. The lines between the components indicate how information on one component is combined with other information in the subsequent step of assessment. Black lines represent major relations while the gray lines illustrate additional data that can be used to underpin specific aspects. The conceptual components are discussed in the following sections.

### System Analysis Taking the Ecological Perspective

The first dimension addresses the status quo of the system in question from an ecological perspective. Here the spatial elements and structures that constitute the GI as well as the functions and services they provide are determined.

#### GI Elements

A broad spectrum of types of green and blue spaces such as nature reserves, agricultural land, woodland, parks, greenways, gardens, allotments, cemeteries, vacant land, wetlands, and all kinds of water bodies is suggested as basic spatial elements of GI (e.g., Davies et al. 2006). In ES research, the spatial elements that deliver ES are named service providing units (SPU) or service providing areas (for a detailed discussion of SPU and related concepts, cf. Wurster and Artmann 2014). For an assessment of multifunctionality that builds on ES, the distinction of GI elements (component 1) should be based on a classification suitable for the analysis of ES.

#### Spatial Relations and GI Networks

Multifunctionality for a GI network needs to take connectivity into account, because connectivity represents the spatial distribution and relations of GI elements and consequently the distribution of benefits they provide (component 2). Connectivity is often referred to as ecological connectivity (e.g., Ahern 2007; Chang et al. 2012). Ecological connectivity is not only meant in a physical sense but also functionally. In the urban matrix, for instance, the distribution of GI elements can impact functions like mitigation of the urban heat island effect, ventilation, and access to green space for recreational use (Pauleit et al. 2011). Here it is suggested to assess connectivity separately for different functions according to the relevance of physical and functional connections.

The spatial dimension of ES is discussed in relation to their provisioning and received benefits. Fisher et al. (2009) distinguished between “service production areas” and “service benefit areas.” They proposed a three-part classification scheme: “in situ” when services are provided in the same location as the benefits received, “omni-directional” when services benefits the surrounding landscape without a specific directional bias, and “directional” if services provided by one area benefit another location. Based on this approach, Syrbe and Walz (2012) suggested to distinguish between “service providing areas,” “service benefiting areas,” and “service connecting areas” that can be mapped. Such frameworks can be used to explore the spatial relations of specific ES and lay the foundation for evidence-based planning of a connected GI network.

#### Supply of ES

An often used example for the classification of ES is promoted by the MA (2005) and TEEB studies (Kumar 2010), which distinguish between provisioning (e.g., food or fresh water), regulating (e.g., local climate regulation), habitat or supporting (e.g., habitats for species), and cultural services (e.g., mental and physical health). Bolund and Hunhammar (1999), Niemelä et al. (2010), and Gómez-Baggethun and Barton (2013) suggest classifications of ES adapted to urban areas.

After deciding which services shall be considered the capacity of GI elements to provide these services is an important component of an assessment of multifunctionality. Supply of ES (component 3) can be understood as the capacity of a particular area to provide these goods and services for which there is an actual demand (Burkhard et al. 2012).

Assessing the supply requires spatial data and appropriate indicators for quantification. For city regions several examples of ES assessments can be found in literature (e.g., Burkhard et al. 2012; Haase et al. 2012), where land cover classes such as those defined by satellite-based CORINE land cover are taken as service providing areas. Measuring units for the assessment are often derived from expert knowledge. Examples for indicators and proxies to quantify the supply ES also have been compiled by de Groot et al. (2010), as well as especially for urban areas by Gómez-Baggethun and Barton (2013). Recommendations for systematic indicator selection are given by van Oudenhoven et al. (2012) and can be used to adapt lists of indicators found in the literature for specific cases.

For quantification of ES not based on measuring units but on relative supply, Burkhard et al. (2012) developed a matrix for linking ES (and ecological integrity indicators) to land cover types. For each land cover type the capacity to provide a particular service was determined based on expert estimations on a scale of 0 (not relevant) to 5 (very high relevant capacity). By linking the matrix within GIS, the spatial distribution of supply could be illustrated (ibid.).

#### Ecosystem Conditions

Emphasizing supply and demand bears the risks of neglecting important properties and processes of ecosystems that are not of immediate or current use but of intermediate or potential use and, moreover, are important for the functioning of the ecosystem. Therefore, Burkhard et al. (2012) developed a conceptual framework of ES supply and demand that includes ecosystem integrity as an overall measure of the system’s condition.

Ecosystem integrity, representing vital ecosystem functions, can be assessed by indicators such as abiotic heterogeneity, biodiversity, or reduction of nutrient loss (ibid. based on Müller 2005). Alternatively, Bastian et al. (2012) suggest including indicators for ecosystem/landscape properties and potentials. Properties should, for example, cover processes of ecosystems/landscape elements and spatial interactions of different elements. Indicators for ecosystem properties (e.g., for land cover and landscape structure, soil, flora, and fauna), functions (e.g., for production functions), and services (e.g., dairy production) have been explored by van Oudenhoven et al. (2012).

Frameworks to integrate a perspective on the condition of ecosystems can also be found in GI literature. Tzoulas et al. (2007) included ecosystem health represented by, for example, air and water quality and ecosystem resilience. Lafortezza et al. (2013) consider biodiversity as a conceptual element in their GI framework.

In line with the above, we recommend that important properties and functions of GI elements not covered by actual supply of ES should be included in a multifunctionality assessment. We, therefore, suggest “condition” of the existing GI elements as a generic term (taking into account concepts like ecosystem integrity and ecosystem health) that can be determined by indicators for specific ecosystem functions or biodiversity and integrated in component 1.

### System Analysis Taking the Social Perspective

The second dimension of the framework takes a social perspective. In ES and GI literature, positive impacts of ES or GI on human well-being such as health benefits are often emphasized (e.g., Tzoulas et al. 2007; Niemelä et al. 2010). However, planning needs to be informed about the actual demand for ES to avoid measures that fail to meet societal needs. Additionally, access to benefits should be considered to prevent unintentional effects that can increase environmental injustice.

#### Demand

GI is often referred to as a collaborative approach that includes local stakeholder perspectives and their demand for GI benefits (component 4). However, the discourses remain on a very general level of acknowledging that social inclusion is an important planning principle (e.g., Kambites and Owen 2006; Pauleit et al. 2011). The question of how to determine demands is still rarely touched. As an exception, Davies et al. (2006) propose standards from green space planning such as ANGST (Accessible Natural Greenspace Standard; Natural England 2010). Such standards define, for example, maximum distances to parks or hectares of local nature reserves per population number that can be transferred into maps and illustrate if the demand is covered.

In ES approaches, demand is crucial because per definition ES do not exist without demand by humans (Fisher et al. 2009). Demand is often determined by expert judgment or politically agreed upon standards. An overview of approaches to derive ES demand such as statistical analysis, modeling, or interviews can be found in Burkhard et al. (2012).

An example for measuring demand on the regional level is given by Kroll et al. (2012). Indicators based on statistical data such as water consumption were used to assess demand of different land cover types (e.g., demand for water per hectare agricultural area). The approach by Burkhard et al. (2012) based on relative values was also applied for assessing demands of ES for different land cover types. The demand for every ES per land cover type was given on a scale ranging from 0 (no relevant demand) to 5 (very high relevant demand). Such approaches can be used to derive spatially explicit representations of the distribution of ES demands at a regional scale based on land cover types and, respectively, GI elements.

Other ES approaches aim at actively including stakeholder groups to derive demands. Diaz et al. (2011) suggest an ES framework that includes stakeholders with direct or indirect claims on land and/or ES. The framework was tested for rural areas with different kind of farmers and conservation agencies as stakeholders. The authors of this study identified together with stakeholder groups how these groups perceive, access, and use ecosystems. Afterward they assessed which priorities the stakeholders have for specific land cover types and the services these provide. Such an approach could be transferred to urban areas. Yet, it needs to be decided for each case whether applying a stakeholder-inclusive approach or an expert-based approach is more adequate.

Furthermore, ecosystem disservices, understood as “functions of ecosystems that are perceived as negative for human well-being,” such as fear stimulated by dense vegetation or damages in gray infrastructure due to growth of tree roots, need to be dealt with (Lyytimaki and Sipila 2009). Concerns articulated by stakeholders related to GI should be considered early in the planning process to avoid conflicts in the subsequent stages.

#### Access to Benefits

Mell (2009) promotes access to green space (component 5) as one major objective for GI planning. Lovell and Taylor (2013) note that GI measures such as greenway or park development and restoration of degraded green and open space such as waterfronts can lead to a displacement of marginalized populations to areas that provide less attractive living conditions than the renewed areas. Distributional impacts are also considered a major social issue for ES implementation since land-use decisions inherently enhance the provision of some ES while reducing others (Robards et al. 2011). Furthermore, access to the benefits from these services may shift between social groups and individuals (Rodríguez et al. 2006).

To operationalize the question of access, Fisher et al. (2009) discuss the public–private good aspect of ES. Services can be rival or non-rival as well as excludable and non-excludable. Rival implies that use of one individual or group reduces the good for others (e.g., crops). Excludable implies that one individual or group can block others from access to an ES (e.g., fruits in a private garden; for further examples and different combinations of private–public goods aspects see ibid.). Such an approach can be a first step to consider the consequences that the enhancement of particular ES can have on societal groups.

### Valuing Multifunctionality

In the third dimension of the approach outlined here, the components for the system analysis are brought together. The valuation determines how the different data set from the analysis can be combined to gain a comprehensive basis for decision-making and priority-setting.

The valuation covers a broad range of approaches, from nominal value scales to decision-support matrixes to less tangible verbal assessments. A synthesis to one aggregated value is not an aim since the effects of normalization (e.g., loss of accuracy) would need to be carefully tested. Additionally, the discourse on advantages and disadvantages of monetary compared to non-monetary valuation in ES (cf., Hein et al. 2006; de Groot et al. 2010; Gómez-Baggethun and Barton 2013) is not integrated because this would exceed the scope of the paper. This does not mean to say that economic approaches could not provide a useful addition to GI planning.

#### GI Integrity

Valuing overall GI integrity is suggested to combine information on GI elements and their conditions (component 1) with that on the spatial relations between them (component 2). The aim is to determine which ecological functions that are relevant for the capacity to supply services of the GI network are crucial for the overall functioning and health of the system.

**Fig. 4:**
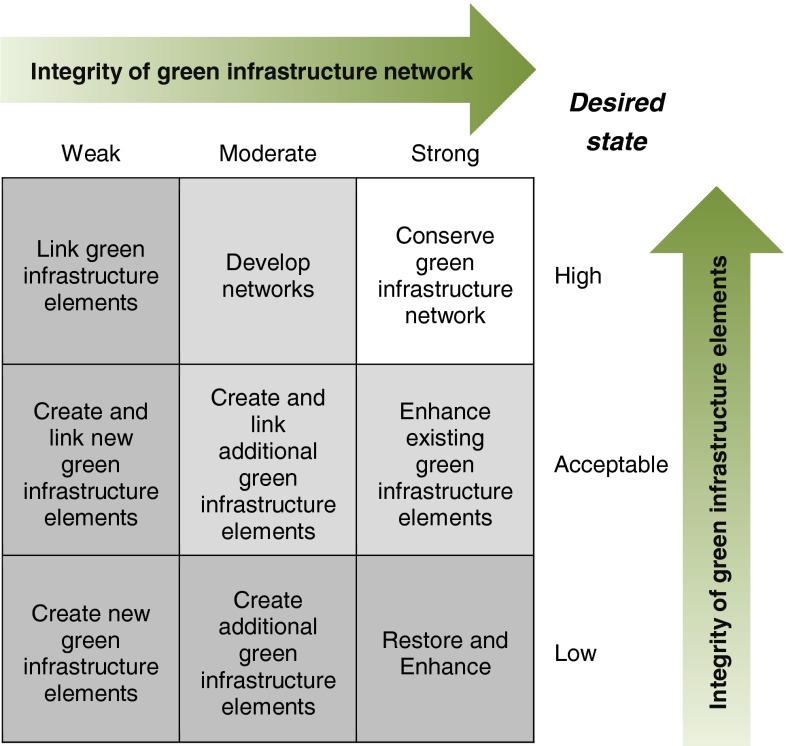
Decision support matrix based on the connectivity of the green infrastructure network and the quality of its elements (adapted from Davies et al. 2006)

Davies et al. (2006) developed a matrix that links the quality of GI elements with the connectivity of the GI network (Fig. 4). Here “quality” is replaced by “integrity” of GI elements. The integrity can be assessed based on indicators presented in the section “ecological conditions” and valued from low to high. Based on data for component 2 the network of GI elements within a particular area can be valued from weak to strong. Such a matrix can be used to derive priorities for improving GI elements as well as the links and gaps between them.

#### Hotspots of Multifunctionality

Services provided by GI elements (component 3) can be not only displayed in separate maps but also summed up to reveal “hotspots” for multifunctionality (component 7). Approaches have been developed to illustrate the overall ES supply of GI elements. Lovell and Taylor (2013) presented a “Multifunctional Landscape Assessment Tool” to survey the performance of ES of small-scale landscape features such as lawns, community gardens, or playgrounds in a park. The value for each service and feature can be added up to an overall performance value for a single green space. For larger areas, The Mersey Forest (2011) developed a city-wide approach (applied for Liverpool) to map functions of GI elements and display how many functions each element provides.

Such tools can reveal which elements provide a high degree of multifunctionality and can be used to explore options for improvements of elements with a lower value. However, priorities for GI improvement based on number of services should not be set without considering synergies and trade-offs between ES (component 8) because increasing particular services should not unintendedly reduce the value for others.

#### Synergies and Trade-Offs

On one hand, the realization of synergies and thus an increase of benefits for humans represent a major objective of GI planning. On the other hand, trade-offs also occur and must be taken into consideration; for instance, conflicts between intensive recreation and the protection of sensitive species from disturbance (Pauleit et al. 2011).

**Fig. 5:**
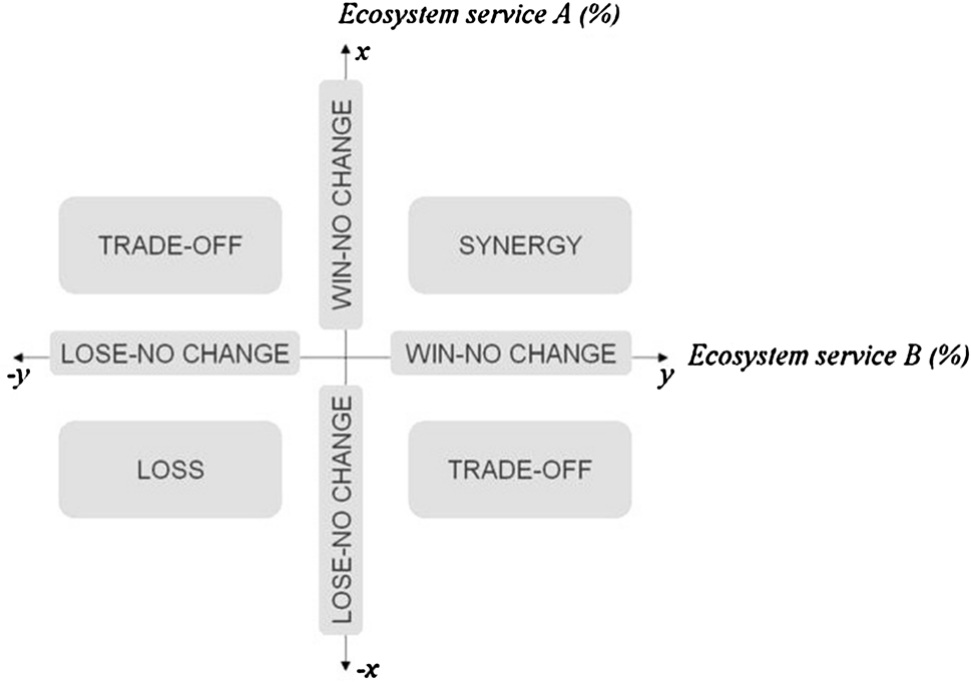
Matrix to determine synergies, trade-offs, and other interrelations between ES (reproduced from Haase et al. 2012 with kind permission by the authors)

Haase et al. (2012) provide a matrix to assess the relation between two ES (Fig. 5). They define a “synergy” as a win–win situation that is determined by an improvement of both ES while a “trade-off” is a loss of one service in exchange for gaining another. “Loss” is a mutual decline in both services. The zero point of either axes represents relations that either improve (“win-no change”) or degrade (“lose-no change”) the provision of one service while the other remains unaffected. Such a matrix can be used to determine synergies and trade-offs of GI strategies (component 8). The relation between important ecological properties and functions should be determined likewise, because otherwise negative effects on GI integrity might be overlooked.

For a comparison of different trade-offs, Rodríguez et al. (2006) suggest a valuation of three factors: spatial scale, temporal scale, and reversibility. The spatial scale is classified in local or large-scale relevance. The temporal scale covers whether a trade-off is of short or long-term effect. Reversibility is determined as reversible or irreversible. In a three-dimensional matrix, trade-offs can then be ordered between least severe (local, short-term effect and reversible) to most severe (large-scale, long-term effect and irreversible). Such a valuation can support decisions between different measures that influence the provision of ES.

#### Supply and Demand

For a comparison of supply and demand (component 9), the data on services provided (component 3) and demand (component 4) are brought together. An important question for the design of such a comparison is if data for supply and demand that have been assessed in the system analysis are comparable. Burkhard et al. (2012) developed an assessment based on matrices of ES and land cover types in which the supply and demand for each service was determined separately for each land cover type and given a rating using a relative scale ranging from 0 to 5. This assessment approach essentially creates relative units for supply and demand of each service. When combined, these values express the supply and demand budgets for each land cover type (e.g., −5 = strong undersupply; 5 = strong oversupply). These values can be transferred to a GIS to spatially reveal the balance of supply and demand for each land cover type. Related to knowledge on flows (which ES can only be experienced “in situ” and which are transferable to other parts; component 2) and questions of access (e.g., maximum distance to recreation areas; component 5), such approaches can inform the overall balance between ES supply and demand of the existing GI and reveal needs for improvement.

#### Stakeholder Preferences

The preferences and interests of different stakeholder groups are often actively elicited in planning processes to aid knowledge transfer and ensure environmental justice. Furthermore, stakeholders can play a vital role as land owners and land managers who can either impede or aid planning decisions, and are thus crucial partners for GI implementation. Therefore, stakeholder preferences hold a separate position in the framework (component 10).

Including stakeholder values requires appropriate methods and detailed knowledge of the case study based on stakeholder insights. Accordingly, engaging stakeholders iteratively is recommended for the identification of crucial ES and values (Chan et al. 2012). The framework by Diaz et al. (2011) already mentioned suggest a weighting by stakeholders: In the step of valuation the information on each stakeholder’s preferences for ES (component 4; also component 5) and the capacity of land cover types to provide those ES (component 3) can be integrated in a multidimensional matrix.

Sanon et al. (2012) developed a multi-criteria decision analysis framework to quantify ES trade-offs for different land-use scenarios of an urban floodplain. They assessed the management objectives different stakeholder groups had for the study area and how stakeholder groups would benefit or be disadvantaged by different wetland restoration scenarios. Such a framework can be used to assess the consequences of specific GI measures, especially from the perspective of different groups of land users such as farmers or fishers.

Such scenario approaches can also be further developed and supported by tools for visualization. For instance, Grêt-Regamey et al. (2013) developed a 3D-GIS modeling environment to illustrate ES trade-offs of three park designs for Masdar City in Abu Dhabi. Photorealistic renderings linked to a visualization of trade-offs can be used to better inform stakeholders on the effects of alternative planning projects.

### Priorities for Strategies and Actions

The last dimension of the assessment is the definition of priorities for GI implementation (component 11). Priorities are understood here as strategies and specific actions that aim at improving the multifunctionality of the GI network. This can include measures for particular GI elements to increase the provision of particular services, to broaden the spectrum of ES provided, or to create new elements where there is a demand. Strategies and actions to close gaps or enhance connectivity in the GI network can also be recommended.

The results for component 6–10 provide the knowledge base that can be used to derive particular strategies and actions. Additionally, best practice studies (e.g., Ahern 2007; Mazza et al. 2011; Pauleit et al. 2011) can inspire GI implementation.

## Discussion

This paper has demonstrated that GI and ES are closely related and may strengthen each other in the development of a common framework for research as well as for implementation. These linkages as well as limitations in the proposed framework for multifunctionality are presented below, along with challenges for mainstreaming the framework in planning and future research to be addressed.

### Possible Linkages Between GI and ES Research

While the concept of ES is still young, its theoretical foundations appear to be already more advanced than GI theory and capable to advance the concept of multifunctionality. Main potentials for integration of the two concepts are seen in the following:

Conceptual frameworks such as the cascade model by Haines-Young and Potschin (2010) or the synergy and trade-off matrix by Haase et al. (2012) can be adapted in GI planning and support a more differentiated consideration of functions, services, and benefits as well as the interrelations between different ES.

Qualitative assessments that, for instance, define ES supply relatively based on expert knowledge can be used to harmonize data from different sources and cover a range of ES (Burkhard et al. 2012; Busch et al. 2012). However, these qualitative assessments are based on proxies and are thus far limited in their precision and scale of application. For instance, the regional-scale indicators tested by Kroll et al. (2012) based on land cover types revealed potential supply and the potential supply–demand ratio but not the actual supply and demand. The more knowledge and relevant indicators developed in the future, the better quantitative approaches will be able to provide more accurate information (e.g., Busch et al. 2012; Bastian et al. 2013).

Next to the assessment of ES provision, approaches for demand have been explored (e.g., Burkhard et al. 2012; Kroll et al. 2012). These approaches can be applied to broaden the GI perspective from demand for recreation to regulating or provisioning services. Additionally, ES approaches that examine demands in cooperation with stakeholder groups (e.g., Diaz et al. 2011) can be adapted to strengthen the social perspective in GI planning. Scenario development can be included in stakeholder group discussions to facilitate an informed discussion (e.g., Ahern 2010; de Groot et al. 2010).

Due to the exponential increase in publications and ongoing high attention to ES, a relatively rapid advancement of theory can be expected. GI planning can on one hand profit from this development. On the other hand, GI research should aim at integrating existing GI concepts and work to strengthen its claims as a distinctive approach to green space planning. For instance, GI contributes a spatial network perspective that can support the determination of spatial relations between ES supply and demand.

### Limitations of the Framework

While there are opportunities for systematically linking the GI and ES concepts, there are also limitations to the suggested framework for multifunctionality which require further discussions. The diverging GI and ES terminology of functions and services is apparent. A broad understanding of functions has the advantage that it can also cover ecosystem properties and processes important for ecological functioning but not of direct use. ES approaches focused on services in direct relation to actual demand might overlook the importance of ecological functioning to secure the long-term capacity to provide services (Bastian et al. 2012).

The integration of ecosystem integrity (Burkhard et al. 2012) or ecosystem health (Tzoulas et al. 2007) as separate assessment components with a set of particular criteria seems suitable to consider ecological conditions. However, Burkhard et al. (2012) note that ecological integrity variables and regulating services inherently overlap. Thus, in assessments double counting or merging of different aspects needs to be considered.

ES classifications such as MA (2005) integrate ecological functioning through the group of supporting services and thus also consider ES for which there is no direct demand. In this regard, testing in case studies is recommendable to get a better picture of advantages and disadvantages of different approaches to integrate ecological functioning in multifunctionality assessments. For implementation in planning practice, research could explore which of these concepts are easier for stakeholders to understand.

Additional limitations occur due to the recent development of ES research and variety of parallel evolving approaches. Assessment standards and widely shared conceptual framework are lacking which hinders comparability and transferability (de Groot et al. 2010).

Several studies from Europe have been applied on a regional scale based on CORINE land cover data (e.g., Haase et al. 2012; Koschke et al. 2012). Not all ES can be adequately assessed based on land cover classes because they depend on particular qualities of GI elements (de Groot et al. 2010). It has to be carefully determined if land cover is an adequate proxy variable for the calculation of various ES and if the resolution of data is detailed enough on a case-by-case basis (Kroll et al. 2012).

Further limitations of the suggested framework occur due to the review approach taken. To narrow the scope of the study the focus lies on literature explicitly related to the concept GI or ES. Other scientific fields such as landscape ecology might provide additional useful methodological elements. For instance, the determination of ecological connectivity could be extended (for a review see, Mazza et al. 2011). An integration of the different components that allows a more structured and comparable valuation such as multi-criteria assessments (e.g., Koschke et al. 2012; Sanon et al. 2012) or Bayesian Belief Networks (cf., de Groot et al. 2010) could also be explored in the future.

Placing a stronger weight on GI in economically driven, cost-oriented decision-making could be furthered by monetary assessment of the multiple benefits GI provides (Mell 2009). The advantages and disadvantages of economic evaluations such as Total Economic Value should be tested in regard to GI (cf., de Groot et al. 2010; Gómez-Baggethun and Barton 2013).

Regarding questions of environmental justice, the assessment of supply and demand proposed in the framework does not capture access to benefits. For example, services provided by a private garden may allow recreation only for a very small group of people, while a larger group still benefits from improved air quality (Ernstson 2013). The theoretical basis for the consideration of undesirable side effects of GI planning, including ecosystem disservices, across social groups also needs to be advanced (Lovell and Taylor 2013).

With regard to stakeholder inclusion, GI and ES approaches will face similar challenges to all stakeholder engagement processes. To build a sound base for decision-making, stakeholder participation needs to be inclusive, legitimate, and informed (cf., Fish 2011). As a tool to empower a local community, for example, Berbés-Blázquez (2012) explored Photovoice, an approach where participants take photos that represent their individual views or views of their social group. Alternative methods could be collaborative mapping with tools such as Public Participation GIS (PPGIS; Brown et al. 2014). Further testing and advancement of these methods could help to strengthen the social perspective in GI planning.

### Implications for Mainstreaming GI Planning

The conceptual framework for multifunctionality proposed here contributes to scientific discourses on GI and ES while supporting the mainstreaming of GI, including informing practitioners how they can design GI planning processes based on the best available knowledge. However, in its complexity the framework might be challenging to implement. Thus, it shall not be viewed as a rigid concept that should be transferred as a whole into planning. Instead, it can and should be tailored for planning tasks on different spatial levels and with particular thematic issues. For example, the methods for analysis need to be adapted according to data availability and, respectively, the capacity to collect data.

A further challenge of GI planning is that it requires knowledge from different professions being brought together, which necessitates establishing new ways of systemic thinking and cross-disciplinary cooperation. Traditional departmental structures in municipalities might hinder such approaches (Kambites and Owen 2006; Primmer and Furman 2012). Interview-based approaches considering institutional processes of learning and adoption of new concepts can help to gain a better understanding of barriers (e.g., Sandström et al. 2006; Niemelä et al. 2010).

What pitfalls and gaps may occur in the implementation of the proposed framework for assessing multifunctionality needs to be explored in case studies. Ahern (2011), for example, advocates project-based collaborations involving various disciplines and adoption of a “Learning-By-Doing” approach. Such approaches based on science–practice cooperation can support the understanding of limitations in practice.

## Conclusion

Planning for multifunctionality aims to create synergies that can be realized in order to increase the overall benefit of GI. However, if multifunctionality would be understood only in a quantitative sense of “the more functions the better,” potential conflicts between different ES might be overlooked. Furthermore, if the capacity of ecosystems to provide services is assessed detached from social questions of demand and access to those benefits, planning for multifunctionality might unintendedly increase environmental injustice for particular groups of society. Thus, multifunctionality needs to be understood as a normative concept and take a broad perspective on urban areas as interrelated social–ecological systems.

From this paper it can be concluded that multifunctionality can be underpinned with a conceptual framework that integrates a broad range of ecological and social aspects and thus meets the holistic thrust of GI planning. The suggested framework for multifunctionality hopefully can foster a discourse on potential linkages and further development of GI and ES approaches. Collaborating more closely could support closing gaps in both concepts. In the future, a combined GI and ES approach could be further developed into innovative planning concept that captures the complexity and dynamic of social–ecological systems in urban areas and supports policy objectives such as sustainable development, environmental justice, social cohesion, or resilience.

## Electronic supplementary material

Below is the link to the electronic supplementary material.

Supplementary material 1 (PDF 202 kb)
