# Supplementary material for: From Multifunctionality to Multiple Ecosystem Services? A Conceptual Framework for Multifunctionality in Green Infrastructure Planning for Urban Areas
Source: Ambio. 2014 Apr 17;43(4):516–29. doi: 10.1007/s13280-014-0510-2 (PMC3989511; doi:10.1007/s13280-014-0510-2)
Supplement: Supplementary file 1 — Supplementary material 1 (PDF 202 kb) [file 13280_2014_510_MOESM1_ESM.pdf]

AMBIO

Electronic Supplementary Material

From multifunctionality to multiple ecosystem services? A conceptual framework for multifunctionality in green infrastructure planning for urban areas

Rieke Hansen and Stephan Pauleit

Appendix S1: List of reviewed publications

Ahern, J. 1995. Greenways as a planning strategy. *Landscape and Urban Planning* 33: 131–155.

Ahern, J. 2007. Green Infrastructure for Cities. The spatial dimension. In *Cities of the future. Towards integrated sustainable water and landscape management,* ed. V. Novotny, 267–283. London: IWA Publ.

Ahern, J. 2010. Planning and Design for Sustainable and Resilient Cities: Theories, Strategies, and Best Practices for Green Infrastructure. In *Water centric sustainable communities. Planning, retrofitting, and building the next urban environment,* ed. V. Novotny, J. Ahern, and P. Brown, 135–176. Hoboken, N.J: John Wiley & Sons.

Alberti, M., J.M. Marzluff, E. Shulenberger, G. Bradley, C. Ryan, and C. Zumbrunnen. 2003. Integrating Humans into Ecology: Opportunities and Challenges for Studying Urban Ecosystems. *BioScience* 53: 1169–1179.

Amati, M., and L. Taylor. 2010. From Green Belts to Green Infrastructure. *Planning Practice and Research* 25:143–155.

Angelstam, P., K. Andersson, R. Axelsson, M. Elbakidze, B. Jonsson, G. Bengt, and J.M. Roberge. 2011. Protecting Forest Areas for Biodiversity in Sweden 1991-2010: the Policy Implementation Process and Outcomes on the Ground. *Silva Fennica* 45: 1111–1133.

Antrop, M. 2006. Sustainable landscapes: contradiction, fiction or utopia? *Landscape and Urban Planning* 75: 187–197.

Axelsson, R., P. Angelstam, E. Degerman, S. Teitelbaum, K. Andersson, M. Elbakidze, and M.K. Drotz. 2013. Social and Cultural Sustainability: Criteria, Indicators, Verifier Variables for Measurement and Maps for Visualization to Support Planning. *AMBIO: A Journal of the Human Environment* 42: 215–228.

Bagstad, K.J., G.W. Johnson, B. Voigt, and F. Villa. 2013. Spatial dynamics of ecosystem service flows: A comprehensive approach to quantifying actual services. *Ecosystem Services* 4:117–125.

Balvanera, P., M. Uriarte, L. Almeida-Leñero, A. Altesor, F. DeClerck, T. Gardner, J. Hall, A. Lara, et al. 2012. Ecosystem services research in Latin America: The state of the art. *Ecosystem Services* 2:56–70.

Barthel, S., and C. Isendahl. 2013. Urban gardens, agriculture, and water management: Sources of resilience for long-term food security in cities. *Ecological Economics* 86: 224–234.

Bastian, O., D. Haase, and K, Grunewald. 2012. Ecosystem properties, potentials and services – The EPPS conceptual framework and an urban application example. *Ecological Indicators* 21: 7–16.

Bastian, O., R.-U. Syrbe, M. Rosenberg, D. Rahe, and K. Grunewald. 2013. The five pillar EPPS framework for quantifying, mapping and managing ecosystem services. *Ecosystem Services* 4: 15–24.

Benedict, M.A., and E.T. McMahon. 2002. Green Infrastructure: Smart Conservation for the 21st Century. *Renewable Resources Journal* 20: 12–17.

Benedict, M.A., and E.T. McMahon. 2006. *Green infrastructure. Linking landscapes and communities.* Washington, DC: Island Press.

Berbés-Blázquez, M. 2012. A Participatory Assessment of Ecosystem Services and Human Wellbeing in Rural Costa Rica Using Photo-Voice. *Environmental Management* 49: 862–875.

Bodurow, C.C., C. Creech, A. Hoback, and J. Martin. 2009. Multivariable Value Densification Modeling Using GIS. *Transactions in GIS* 13:147–175.

Bolund, P., and S. Hunhammar. 1999. Ecosystem services in urban areas. *Ecological Economics* 29: 293–301.

Boyd, J., and S. Banzhaf. 2007. What are ecosystem services? The need for standardized environmental accounting units. *Ecological Economics* 63: 616–626.

Braat, L. C., and R. de Groot. 2012. The ecosystem services agenda: bridging the worlds of natural science and economics, conservation and development, and public and private policy. *Ecosystem Services* 1: 4–15.

Brown, G., M.F. Schebella, and D. Weber. 2014. Using participatory GIS to measure physical activity and urban park benefits. *Landscape and Urban Planning* 121: 34–44.

Bryant, M.M. 2006. Urban landscape conservation and the role of ecological greenways at local and metropolitan scales. *Landscape and Urban Planning* 76: 23–44.

Burkhard, B., F. Kroll, S. Nedkov, and F. Müller. 2012. Mapping ecosystem service supply, demand and budgets. *Ecological Indicators* 21: 17–29.

Burkhard, B., N. Crossman, S. Nedkov, K. Petz, and R. Alkemade. 2013. Mapping and modelling ecosystem services for science, policy and practice. *Ecosystem Services* 4: 1–3.

Burkhard, B., R.S. de Groot, R. Costanza, R. Seppelt, S.E. Jørgensen, and M. Potschin. 2012. Solutions for sustaining natural capital and ecosystem services. *Ecological Indicators* 21: 1–6.

Busch, M., A. La Notte, V. Laporte, and M. Erhard. 2012. Potentials of quantitative and qualitative approaches to assessing ecosystem services. *Ecological Indicators* 21: 89–103.

Butler, C.D., and W. Oluoch-Kosura. 2006. Linking Future Ecosystem Services and Future Human Well-being. *Ecology and Society* 11: 30.

Carpenter, S.R., E.M. Bennett, and G.D. Peterson. 2006. Scenarios for Ecosystem Services: An Overview. *Ecology and Society* 11:29.

Carpenter, S.R., H.A. Mooney, J. Agard, D. Capistrano, R.S. DeFries, S. Diaz, T. Dietz, A.K. Duraiappah, et al. 2009. Science for managing ecosystem services. Beyond the Millennium Ecosystem Assessment. *Proceedings of the National Academy of Sciences* 106: 1305–1312.

Chan, K.M.A., A.D. Guerry, P. Balvanera, S. Klain, T. Satterfield, X. Basurto, A. Bostrom, R. Chuenpagdee, et al. 2012. Where are Cultural and Social in Ecosystem Services? A Framework for Constructive Engagement. *BioScience* 62:744–756.

Chan, K.M.A., M.R. Shaw, D.R. Cameron, E.C. Underwood, and G.C. Daily. 2006. Conservation Planning for Ecosystem Services. *PLoS Biology* 4:e379.

Chan, K.M.A., T. Satterfield, and J. Goldstein. 2012. Rethinking ecosystem services to better address and navigate cultural values. *Ecological Economics* 74: 8–18.

Chang, Q., X. Li, X. Huang, and J. Wu. 2012. A GIS-based Green Infrastructure Planning for Sustainable Urban Land Use and Spatial Development. *Procedia Environmental Sciences* 12: 491–498.

Cowling, R.M., B. Egoh, A.T. Knight, P.J. O'Farrell, B. Reyers, M. Rouget, D. J. Roux, A. Welz, and A. Wilhelm-Rechman. 2008. Ecosystem Services Special Feature: An operational model for mainstreaming ecosystem services for implementation. *Proceedings of the National Academy of Sciences* 105: 9483–9488.

Crossman, N.D., B. Burkhard, S. Nedkov, L. Willemen, K. Petz, I. Palomo, E. G. Drakou, B. Martín-Lopez, et al. 2013. A blueprint for mapping and modelling ecosystem services. *Ecosystem Services* 4: 4–14.

Daniel, T.C., A. Muhar, A. Arnberger, O. Aznar, J.W. Boyd, K.M.A. Chan, R. Costanza, T. Elmqvist, et al. 2012. Contributions of cultural services to the ecosystem services agenda. *Proceedings of the National Academy of Sciences* 109: 8812–8819.

Davies, C., R. MacFarlane, C. McGloin, and M. Roe. 2006. *Green Infrastructure Planning Guide.*

Daw, T., K. Brown, S. Rosendo, and R. Pomeroy. 2011. Applying the ecosystem services concept to poverty alleviation: the need to disaggregate human well-being. *Environmental Conservation* 38: 370–379.

Groot, R. de. 2006. Function-analysis and valuation as a tool to assess land use conflicts in planning for sustainable, multi-functional landscapes. *Landscape and Urban Planning* 75: 175–186.

Diaz, S., F. Quetier, D.M. Caceres, S.F. Trainor, N. Perez-Harguindeguy, M.S. Bret-Harte, B. Finegan, M. Pena-Claros, et al. 2011. Linking functional diversity and social actor strategies in a framework for interdisciplinary analysis of nature's benefits to society. *Proceedings of the National Academy of Sciences* 108: 895–902.

Dobbs, C., F.J. Escobedo, and W.C. Zipperer. 2011. A framework for developing urban forest ecosystem services and goods indicators. *Landscape and Urban Planning* 99: 196–206.

Egoh, B.N., P.J. O'Farrell, A. Charef, L.J. Gurney, T. Koellner, H. Nibam Abi, M. Egoh, and L. Willemen. 2012. An African account of ecosystem service provision: Use, threats and policy options for sustainable livelihoods. *Ecosystem Services* 2: 71–81.

Ernstson, H. 2013. The social production of ecosystem services: A framework for studying environmental justice and ecological complexity in urbanized landscapes. *Landscape and Urban Planning* 109: 7–17.

Ernstson, H., and S. Sörlin. 2012. Ecosystem services as technology of globalization: On articulating values in urban nature. *Ecological Economics* 86: 274–284.

European Commission 2013. Communication from the Commission to the European Parliament, the council, the European Economic and Social Committee and the Committee of the Regions. *Green Infrastructure (GI) — Enhancing Europe's Natural Capital*. COM(2013) 249 final.

Fabos, J.G., and R.L. Ryan. 2006. An introduction to greenway planning around the world. *Landscape and Urban Planning* 76: 1–6.

Faith, D P., S. Magallón, A.P. Hendry, E. Conti, T. Yahara, and M.J. Donoghue. 2010. Ecosystem services: an evolutionary perspective on the links between biodiversity and human well-being. *Current Opinion in Environmental Sustainability* 2: 66–74.

Fish, R.D. 2011. Environmental decision making and an ecosystems approach: Some challenges from the perspective of social science. *Progress in Physical Geography* 35: 671–680.

Fisher, B., R.K. Turner, and P. Morling. 2009. Defining and classifying ecosystem services for decision making. *Ecological Economics* 68: 643–653.

Frischenbruder, M.T.M., and P. Pellegrino. 2006. Using greenways to reclaim nature in Brazilian cities. *Landscape and Urban Planning* 76: 67–78.

Gallent, N., and D. Shaw. 2007. Spatial planning, area action plans and the rural-urban fringe. *Journal of Environmental Planning and Management* 50:617–638.

Gill, S.E., J.F. Handley, A.R. Ennos, S. Pauleit, N. Theuray, and S.J. Lindley. 2008. Characterising the urban environment of UK cities and towns: A template for landscape planning. *Landscape and Urban Planning* 87: 210–222.

Gómez-Baggethun, E., and D.N. Barton. 2013. Classifying and valuing ecosystem services for urban planning. *Ecological Economics* 86: 235–245.

Gomez-Baggethun, E., R. de Groot, P.L. Lomas, and C. Montes. 2010. The history of ecosystem services in economic theory and practice: From early notions to markets and payment schemes. *Ecological Economics* 69:1209–1218.

Gordon, A., D. Simondson, M. White, A. Moilanen, and S.A. Bekessy. 2009. Integrating conservation planning and landuse planning in urban landscapes. *Landscape and Urban Planning* 91: 183–194.

Grant, L. E. 2012. Briefing: Making space for green places. *Proceedings of the Institution of Civil Engineers–Engineering Sustainability* 165: 121–123.

Grêt-Regamey, A., E. Celio, T.M. Klein, and U. Wissen Hayek. 2013. Understanding ecosystem services trade-offs with interactive procedural modeling for sustainable urban planning. *Landscape and Urban Planning* 109: 107–116.

Groot, R.S. de, R. Alkemade, L. Braat, L. Hein, and L. Willemen. 2010. Challenges in integrating the concept of ecosystem services and values in landscape planning, management and decision making. *Ecological Complexity* 7: 260–272.

Guimarães, M H., J. Ballé-Béganton, D. Bailly, A. Newton, T. Boski, and T. Dentinho. 2013. Transdisciplinary conceptual modeling of a social-ecological system – A case study application in Terceira Island, Azores. *Ecosystem Services* 3: e22-e31.

Haaren, C. von, and C. Albert. 2011. Integrating ecosystem services and environmental planning: limitations and synergies. *International Journal of Biodiversity Science, Ecosystem Services & Management* 7: 150–167.

Haaren, C. von, and M. Reich. 2006. The German way to greenways and habitat networks. *Landscape and Urban Planning* 76: 7–22.

Haase, D., N. Larondelle, T. McPhearson, N. Schwarz, Z. Hamstead, P. Kremer, M. Artmann, D. Wurster, et al. 2014. A quantitative review of urban ecosystem services assessment: concepts, models and implementation. In *AMBIO: A Journal of the Human Environment*: this issue.

Haase, D., N. Schwarz, M. Strohbach, F. Kroll, and R. Seppelt. 2012. Synergies, Trade-offs, and Losses of Ecosystem Services in Urban Regions: an Integrated Multiscale Framework Applied to the Leipzig-Halle Region, Germany. *Ecology and Society* 17: 22.

Haines-Young, R., and M. Potschin 2010. The links between biodiversity, ecosystem services and human well-being. In *Ecosystem ecology. A new synthesis*, ed. C. Frid and D.G. Raffaelli, 110–139. New York: Cambridge University Press.

Haines-Young, R., M. Potschin, and F. Kienast. 2012. Indicators of ecosystem service potential at European scales: Mapping marginal changes and trade-offs. *Ecological Indicators* 21: 39–53.

Handley, J., S. Pauleit, S. and S. Gill. 2007. Landscape sustainability and the city. In *Landscape and Sustainability,* ed. J.F. Benson, and M. Roe, 167-195. Routledge, London:

Hauck, J., C. Görg, R. Varjopuro, O. Ratamäki, and K. Jax. 2013. Benefits and limitations of the ecosystem services concept in environmental policy and decision making: Some stakeholder perspectives. *Environmental Science and Policy* 25:13–21.

Hauck, J., C. Görg, R. Varjopuro, O. Ratamäki, J. Maes, H. Wittmer, and K. Jax. 2013. "Maps have an air of authority": Potential benefits and challenges of ecosystem service maps at different levels of decision making. *Ecosystem Services* 4:25–32.

Hein, L., K. van Koppen, R.S. de Groot, and E.C. van Ierland. 2006. Spatial scales, stakeholders and the valuation of ecosystem services. *Ecological Economics* 57: 209–228.

Hofmann, M., J.R. Westermann, I. Kowarik, and E. van der Meer. 2012. Perceptions of parks and urban derelict land by landscape planners and residents. *Urban Forestry and Urban Greening* 11: 303–312.

Horwood, K. 2011. Green infrastructure: reconciling urban green space and regional economic development: lessons learnt from experience in England's north-west region. *Local Environment* 16: 963–975.

Hostetler, M., W. Allen, and C. Meurk. 2011. Conserving urban biodiversity? Creating green infrastructure is only the first step. *Landscape and Urban Planning* 100: 369–371.

Hubacek, K., and J. Kronenberg. 2013. Synthesizing different perspectives on the value of urban ecosystem services. *Landscape and Urban Planning* 109: 1–6.

Hunter, M.C.R., and D.G. Brown. 2012. Spatial contagion: Gardening along the street in residential neighborhoods. *Landscape and Urban Planning* 105: 407–416.

Ignatieva, M., and K. Ahrné. 2013. Biodiverse green infrastructure for the 21st century: from "green desert" of lawns to biophilic cities. *Journal of Architecture and Urbanism* 37: 1–9.

James, P., K. Tzoulas, M. Adams, A. Barber, J. Box, J. Breuste, T. Elmqvist, M. Frith, et al. 2009. Towards an integrated understanding of green space in the European built environment. *Urban Forestry and Urban Greening* 8: 65–75.

Jim, C., and S.S. Chen. 2003. Comprehensive greenspace planning based on landscape ecology principles in compact Nanjing city, China. *Landscape and Urban Planning* 65: 95–116.

Jones-Walters, L., and A. Çil. 2011. Biodiversity and stakeholder participation. *Journal for Nature Conservation* 19: 327–329.

Jongman, R.H. 1995. Nature conservation planning in Europe: developing ecological networks. *Landscape and Urban Planning* 32: 169–183.

Jongman, R.H., M. Külvik, and I. Kristiansen. 2004. European ecological networks and greenways. *Landscape and Urban Planning* 68: 305–319.

Jordan, S.J., S.E. Hayes, D. Yoskowitz, L.M. Smith, J.K. Summers, M. Russell, and W.H. Benson. 2010. Accounting for Natural Resources and Environmental Sustainability: Linking Ecosystem Services to Human Well-Being. *Environmental Science and Technology* 44:1530–1536.

Jørgensen, S.E., and S.N. Nielsen. 2012. Tool boxes for an integrated ecological and environmental management. *Ecological Indicators* 21: 104–109.

Kambites, C., and S. Owen. 2006. Renewed prospects for green infrastructure planning in the UK. *Planning Practice and Research* 21: 483–496.

Keeler, B L., S. Polasky, K.A. Brauman, K.A. Johnson, J.C. Finlay, A. O'Neill, K. Kovacs, and B. Dalzell. 2012. Linking water quality and well-being for improved assessment and valuation of ecosystem services. *Proceedings of the National Academy of Sciences* 109: 18619–18624.

Keeley, M. 2011. The Green Area Ratio: an urban site sustainability metric. *Journal of Environmental Planning and Management* 54: 937–958.

Kennedy, C., S. Pincetl, and P. Bunje. 2011. The study of urban metabolism and its applications to urban planning and design. *Environmental Pollution* 159: 1965–1973.

Kenward, R.E., M.J. Whittingham, S. Arampatzis, B.D. Manos, T. Hahn, A. Terry, R. Simoncini, J. Alcorn, et al. 2011. Identifying governance strategies that effectively

support ecosystem services, resource sustainability, and biodiversity. *Proceedings of the National Academy of Sciences* 108: 5308–5312.

Kilbane, S. 2013. Green infrastructure: planning a national green network for Australia. *Journal of Landscape Architecture* 8: 64–73.

Kithiia, J., and A. Lyth. 2011. Urban wildscapes and green spaces in Mombasa and their potential contribution to climate change adaptation and mitigation. *Environment and Urbanization* 23: 251–265.

Koschke, L., C. Fürst, S. Frank, and F. Makeschin. 2012. A multi-criteria approach for an integrated land-cover-based assessment of ecosystem services provision to support landscape planning. *Ecological Indicators* 21: 54–66.

Kroll, F., F. Müller, D. Haase, and N. Fohrer. 2012. Rural–urban gradient analysis of ecosystem services supply and demand dynamics. *Land Use Policy* 29: 521–535.

Kumar, P. 2010. *The economics of ecosystems and biodiversity. Ecological and economic foundations. [*TEEB: The Economics of Ecosystems and Biodiversity]. London [u.a.]: Earthscan.

La Greca, P., D. La Rosa, F. Martinico, and R. Privitera. 2011. Agricultural and green infrastructures: The role of non-urbanised areas for eco-sustainable planning in a metropolitan region. *Environmental Pollution* 159: 2193–2202.

La Rosa, D., and R. Privitera. 2013. Characterization of non-urbanized areas for land-use planning of agricultural and green infrastructure in urban contexts. *Landscape and Urban Planning* 109: 94–106.

Lafortezza, R., C. Davies, G. Sanesi, and C.C. Konijnendijk. 2013. Green Infrastructure as a tool to support spatial planning in European urban regions. *iForest* 6: 102–108.

Larondelle, N., and D. Haase. 2013. Urban ecosystem services assessment along a rural–urban gradient: A cross-analysis of European cities. *Ecological Indicators* 29: 179–190.

Larson, E., and C. Perrings. 2013. The value of water-related amenities in an arid city: The case of the Phoenix metropolitan area. *Landscape and Urban Planning* 109: 45–55.

Li, Y., Y. Li, H. Zhang, Y. Liu, W. Xu, and X. Zhu. 2011. Canadian experience in low carbon eco-city development and the implications for China. *Energy Procedia* 5: 1791–1795.

Linehan, J. G. M. F. J. 1995. Greenway planning: Developing a landscape ecological network approach. *Landscape and Urban Planning* 33: 179.

Liu, S., R. Costanza, S. Farber, and A. Troy. 2010. Valuing ecosystem services. *Annals of the New York Academy of Sciences* 1185: 54–78.

Llausas, A., and M. Roe. 2012. Green Infrastructure Planning: Cross-National Analysis between the North East of England (UK) and Catalonia (Spain). In *European Planning Studies* 20: 641–663.

Lovell, S.T., and J.R. Taylor. 2013. Supplying urban ecosystem services through multifunctional green infrastructure in the United States. *Landscape Ecology* 28: 1447-1463.

Luck, G.W., K.M.A. Chan, U. Eser, E. Gómez-Baggethun, B. Matzdorf, B. Norton, and M. Potschin. 2012. Ethical Considerations in On-Ground Applications of the Ecosystem Services Concept. *BioScience* 62: 1020–1029.

Lyytimaki, J., and M. Sipila. 2009. Hopping on one leg - The challenge of ecosystem disservices for urban green management. *Urban Forestry and Urban Greening* 8: 309–315.

MA 2005. *Ecosystems and human well-being*. Synthesis. A report of the Millennium Ecosystem Assessment. Washington, DC: Island Press.

Madureira, H., T. Andresen, and A. Monteiro. 2011. Green structure and planning evolution in Porto. *Urban Forestry and Urban Greening* 10:141–149.

Maes, J., B. Egoh, L. Willemen, C. Liquete, P. Vihervaara, J.P. Schägner, B. Grizzetti, E.G. Drakou, et al. 2012. Mapping ecosystem services for policy support and decision making in the European Union. *Ecosystem Services* 1: 31–39.

Mansor, M., I. Said, and I. Mohamad. 2012. Experiential Contacts with Green Infrastructure's Diversity and Well-being of Urban Community. *Social and Behavioral Sciences* 49: 257–267.

Marcucci, D.J., and L.M. Jordan. 2013. Benefits and Challenges of Linking Green Infrastructure and Highway Planning in the United States. *Environmental Management* 51: 182–197.

Mariarinaldi, B. 2007. Landscapes of metropolitan hedonism The Cheonggyecheon Linear Park in Seoul. *Journal of Landscape Architecture* 2: 60–73.

Mascarenhas, A., T.B. Ramos, and L. Nunes. 2012. Developing an integrated approach for the strategic monitoring of regional spatial plans. *Land Use Policy* 29: 641–651.

Mazza, L., G. Bennett, L. de Nocker, S. Gantioler, L. Losarcos, C. Margerison, T. Kaphengst, A. McConville, et al. 2011. *Green Infrastructure Implementation and Efficiency.* Final report for the European Commission, DG Environment on Contract ENV.B.2/SER/2010/0059. Institute for European Environmental Policy. Brussels and London.

McDonald, L.A., W.L. Allen, M.A. Benedict, and K. O'Conner. 2005. Green Infrastructure Plan Evaluation Frameworks. *Journal of Conservation Planning* 1: 6–25.

McLain, R., M. Poe, P.T. Hurley, J. Lecompte-Mastenbrook, and M. R. Emery. 2012. Producing edible landscapes in Seattle's urban forest. *Urban Forestry and Urban Greening* 11: 187–194.

McPhearson, T., P. Kremer, and Z.A. Hamstead. 2013. Mapping ecosystem services in New York City: Applying a social–ecological approach in urban vacant land. *Ecosystem Services* 5: 11–26.

McWilliam, W.J., P.F.J. Eagles, M.L. Seasons, and R.D. Brown. 2012. Effectiveness of Boundary Structures in Limiting Residential Encroachment into Urban Forests. *Landscape Research* 37: 301–325.

McWilliam, W., P. Eagles, M. Seasons, and R. Brown. 2010. The housing-forest interface: Testing structural approaches for protecting suburban natural systems following development. *Urban Forestry and Urban Greening* 9: 149–159.

Mell, I.C. 2009. Can green infrastructure promote urban sustainability? *Proceedings of the Institution of Civil Engineers–Engineering Sustainability* 162: 23–34.

Mell, I.C. 2013. Can you tell a green field from a cold steel rail? Examining the "green" of Green Infrastructure development. *Local Environment* 18: 152–166.

Messer, K.D. 2006. The conservation benefits of cost-effective land acquisition: A case study in Maryland. *Journal of Environmental Management 79*: 305–315.

Mitsova, D., W. Shuster, and X. Wang. 2011. A cellular automata model of land cover change to integrate urban growth with open space conservation. *Landscape* and Urban Planning 99: 141–153.

Molnar, J.L., and I. Kubiszewski. 2012. Managing natural wealth: Research and implementation of ecosystem services in the United States and Canada. *Ecosystem Services* 2: 45–55.

Moseley, D., M. Marzano, J. Chetcuti, and K. Watts. 2013. Green networks for people: Application of a functional approach to support the planning and management of greenspace. *Landscape and Urban Planning* 116: 1–12.

Müller, F. 2005. Indicating ecosystem and landscape organisation. *Ecological Indicators* 5: 280–294.

Müller, F., and B. Burkhard. 2012. The indicator side of ecosystem services. *Ecosystem Services* 1: 26–30.

Muradian, R., and L. Rival. 2012. Between markets and hierarchies: The challenge of governing ecosystem services. *Ecosystem Services* 1: 93–100.

Natural England. 2010. *Nature Nearby. Accessible Natural Greenspace Guidance.* Retrieved 31 November, 2013, from www.naturalengland.org.uk.

Naumann, S., M. Davis, T. Kaphengst, M. Pieterse, and M. Rayment. 2011. *Design, implementation and cost elements of Green Infrastructure projects.* Final report to the European Commission, DG Environment, Contract no. 070307/2010/577182/ETU/F.1.

Nedkov, S. &. B. Burkhard. 2012. Flood regulating ecosystem services - Mapping supply and demand, in the Etropole municipality, Bulgaria. *Ecological Indicators* 21: 67–79.

Nemec, K.T., and C. Raudsepp-Hearne. 2013. The use of geographic information systems to map and assess ecosystem services. *Biodiversity and Conservation* 22: 1–15.

Nickel, D., W. Schoenfelder, D. Medearis, D.P. Dolowitz, M. Keeley, and W. Shuster. 2013. German experience in managing stormwater with green infrastructure. *Journal of Environmental Planning and Management*: 1–21.

Niemelä, J., S.R. Saarela, T. Soderman, L. Kopperoinen, V. Yli-Pelkonen, S. Vare, and D.J. Kotze. 2010. Using the ecosystem services approach for better planning and

conservation of urban green spaces. A Finland case study. Biodiversity and Conservation 19: 3225–3243.

Norgaard, R B. 2010. Ecosystem services: From eye-opening metaphor to complexity blinder. *Ecological Economics* 69: 1219–1227.

Novotny, V., J. Ahern, and P. Brown, ed. 2010. *Water centric sustainable communities. Planning, retrofitting, and building the next urban environment.* John Wiley & Sons, Hoboken, N.J.

Opermanis, O., B. MacSharry, A. Aunins, and Z. Sipkova. 2012. Connectedness and connectivity of the Natura 2000 network of protected areas across country borders in the European Union. *Biological Conservation* 153: 227–238.

Palang, H., T. Spek, and M. Stenseke. 2011. Digging in the past: New conceptual models in landscape history and their relevance in peri-urban landscapes. *Landscape and Urban Planning* 100: 344–346.

Palomo, I., B. Martín-López, M. Potschin, R. Haines-Young, and C. Montes. 2013. National Parks, buffer zones and surrounding lands: Mapping ecosystem service flows. *Ecosystem Services* 4: 104–116.

Paloniemi, R., E. Apostolopoulou, E. Primmer, M. Grodzinska-Jurcak, K. Henle, I. Ring, M. Kettunen, J. Tzanopoulos, S. Potts, S. van den Hove, P. Marty, A. McConville, and J. Simila. 2012. Biodiversity conservation across scales: lessons from a science–policy dialogue. *Nature Conservation* 2: 7–19.

Pataki, D.E., M.M. Carreiro, J. Cherrier, N.E. Grulke, V. Jennings, S. Pincetl, R.V. Pouyat, T.H. Whitlow, and W.C. Zipperer. 2011. Coupling biogeochemical cycles in urban environments: ecosystem services, green solutions, and misconceptions. *Frontiers in Ecology and the Environment* 9: 27–36.

Pauleit, S.. L. Liu, J. Ahern, and A. Kazmierczak. 2011. Multifunctional green infrastructure planning to promote ecological services in the city. In *Urban ecology. Patterns, processes, and applications,* ed. J. Niemelä, 272–285. Oxford: Oxford Univ. Press.

Pickett, S.T.A., M.L. Cadenasso, J.M. Grove, C.G. Boone, P.M. Groffman, E. Irwin, S.S. Kaushal, V. Marshall, et al. 2011. Urban ecological systems: Scientific foundations and a decade of progress. *Journal of Environmental Management* 92: 331–362.

Pittock, J., S. Cork, and S. Maynard. 2012. The state of the application of ecosystems services in Australia. *Ecosystem Services* 1: 111–120.

Piwowarczyk, J., J. Kronenberg, and M.A. Dereniowska. 2013. Marine ecosystem services in urban areas: Do the strategic documents of Polish coastal municipalities reflect their importance? *Landscape and Urban Planning* 109: 85–93.

Polishchuk, Y., and F. Rauschmayer. 2012. Beyond "benefits"? Looking at ecosystem services through the capability approach. *Ecological Economics* 81: 103–111.

Potschin, M., and R. Haines-Young. 2006. "Rio+10", sustainability science and Landscape Ecology. *Landscape and Urban Planning* 75: 162–174.

Primmer, E. and E. Furman. 2012. Operationalising ecosystem service approaches for governance: Do measuring, mapping and valuing integrate sector-specific knowledge systems? *Ecosystem Services* 1: 85–92.

Qureshi, S., S.J.H. Kazmi, and J.H. Breuste. 2010. Ecological disturbances due to high cutback in the green infrastructure of Karachi: Analyses of public perception about associated health problems. Urban Forestry & Urban Greening 9: 187–198.

Randolph, J. 2012. *Environmental land use planning and management*. Island Press, Washington.

Raudsepp-Hearne, C., G.D. Peterson, M. Tengö, E.M. Bennett, T. Holland, K. Benessaiah, G. K. MacDonald, and L. Pfeifer. 2010. Untangling the Environmentalist's Paradox: Why Is Human Well-being Increasing as Ecosystem Services Degrade? *BioScience* 60: 576–589.

Robards, M.D., M.L. Schoon, C.L. Meek, and N.L. Engle. 2011. The importance of social drivers in the resilient provision of ecosystem services. *Global Environmental Change* 21: 522–529.

Rodríguez, J.P., T. Beard, J.R. Douglas, E.M. Bennett, G.S. Cumming, S.J. Cork, J. Agard, A.P. Dobson, et al. 2006. Trade-offs across Space, Time, and Ecosystem Services. *Ecology and Society* 11: 28.

Ruliffson, J.A., P.H. Gobster, R.G. Haight, and F.R. Homans. 2002. Niches in the urban forest: Organizations and their role in acquiring metropolitan open space. *Journal of Forestry* 100: 16–23.

Sandström, U.G. 2002. Green Infrastructure Planning in Urban Sweden. *Planning Practice and Research* 17: 373–385.

Sandström, U.G., P. Angelstam, and A. Khakee. 2006. Urban comprehensive planning – identifying barriers for the maintenance of functional habitat networks. *Landscape and Urban Planning* 75: 43–57.

Sanon, S., T. Hein, W. Douven, and P. Winkler. 2012. Quantifying ecosystem service trade-offs: The case of an urban floodplain in Vienna, Austria. *Journal of Environmental Management* 111: 159–172.

Schäffler, A., and M. Swilling. 2013. Valuing green infrastructure in an urban environment under pressure — The Johannesburg case. *Sustainable Urbanisation: A resilient future* 86: 246–257.

Schetke, S., and D. Haase. 2008. Multi-criteria assessment of socio-environmental aspects in shrinking cities. Experiences from eastern Germany. *Environmental Impact Assessment Review* 28: 483–503.

Schetke, S., D. Haase, and T. Kötter. 2012. Towards sustainable settlement growth: A new multi-criteria assessment for implementing environmental targets into strategic urban planning. *Environmental Impact Assessment Review* 32: 195–210.

Schetke, S., D. Haase, and J.H. Breuste. 2010. Green space functionality under conditions of uneven urban land use development. *Journal of Land Use Science* 5: 143–158.

Schilling, J., and J. Logan. 2008. Greening the Rust Belt A Green Infrastructure Model for Right Sizing America's Shrinking Cities. *Journal of the American Planning Association*: 451–466.

Schneiders, A., T. van Daele, W. van Landuyt, and W. van Reeth. 2012. Biodiversity and ecosystem services: Complementary approaches for ecosystem management? *Ecological Indicators* 21: 123–133.

Schrijnen, P.M. (2000): Infrastructure networks and red-green patterns in city regions. *Landscape and Urban Planning* 48: 191–204.

Schwarz, N., A. Bauer, and D. Haase. 2011. Assessing climate impacts of planning policies—An estimation for the urban region of Leipzig (Germany). *Environmental Impact Assessment Review* 31: 97–111.

Seppelt, R., B. Fath, B. Burkhard, J.L. Fisher, A. Grêt-Regamey, S. Lautenbach, P. Pert, S. Hotes, et al. 2012. Form follows function? Proposing a blueprint for ecosystem service assessments based on reviews and case studies. *Ecological Indicators* 21: 145–154.

Seppelt, R., C.F. Dormann, F.V. Eppink, S. Lautenbach, and S. Schmidt. 2011. A quantitative review of ecosystem service studies: approaches, shortcomings and the road ahead. *Journal of Applied Ecology* 48: 630–636.

Stubbs, M. 2008. Natural green space and planning policy: Devising a model for its delivery in regional spatial strategies. *Landscape Research* 33: 119–139.

Summers, J.K., L.M. Smith, J.L. Case, and R.A. Linthurst. 2012. A Review of the Elements of Human Well-Being with an Emphasis on the Contribution of Ecosystem Services. *AMBIO: A Journal of the Human Environment* 41: 327–340.

Syrbe, R.-U. and U. Walz. 2012. Spatial indicators for the assessment of ecosystem services: Providing, benefiting and connecting areas and landscape metrics. *Ecological Indicators* 21: 80–88.

TEEB – The Economics of Ecosystems and Biodiversity. 2011. *TEEB Manual for Cities: Ecosystem Services in Urban Management.*

The Mersey Forest. 2011. *The Value of Mapping Green Infrastructure.* Retrieved 17 November, 2013, from www.merseyforest.org.uk/files/The_Value_of_Mapping_Green_Infrastructure_pdf.pdf.

The North West Green Infrastructure Think Tank. 2008. *North West Green Infrastructure Guide.* Version 1.1. Retrieved 6 November, 2013, from www.greeninfrastructurenw.co.uk/resources/GIguide.pdf.

Thomas, K., and S. Littlewood. 2010. From Green Belts to Green Infrastructure? The Evolution of a New Concept in the Emerging Soft Governance of Spatial Strategies. *Planning Practice and Research* 25: 203–222.

Turner, T. 1996. *City as landscape. A post-modern view of design and planning.* E & FN Spon, London.

Turner, T. 2006. Greenway planning in Britain: recent work and future plans. *Landscape and Urban Planning* 76: 240–251.

Tzoulas, K., and P. James. 2010. Peoples' use of, and concerns about, green space networks: A case study of Birchwood, Warrington New Town, UK. Urban *Forestry and Urban Greening* 9: 121–128.

Tzoulas, K., K. Korpela, S. Venn, V. Yli-Pelkonen, A. Kazmierczak, J. Niemelä, and P. James. 2007. Promoting ecosystem and human health in urban areas using Green Infrastructure. A literature review. *Landscape and Urban Planning* 81: 167–178.

Underwood, J.G., J. Francis, and L.R. Gerber. 2011. Incorporating biodiversity conservation and recreational wildlife values into smart growth land use planning. *Landscape and Urban Planning* 100:136–143.

van Oudenhoven, A.P.E., K. Petz, R. Alkemade, L. Hein, and R.S. de Groot. 2012. Framework for systematic indicator selection to assess effects of land management on ecosystem services. *Ecological Indicators* 21: 110–122.

Vandermeulen, V., A. Verspecht, B. Vermeire, G. van Huylenbroeck, and X. Gellynck. 2011. The use of economic valuation to create public support for green infrastructure investments in urban areas. *Landscape and Urban Planning 103*: 198–206.

Wallace, K.J. 2007. Classification of ecosystem services: Problems and solutions. *Biological Conservation* 139: 235–246.

Walmsley, A. 1995. Greenways and the making of urban form. *Landscape and Urban Planning* 33: 81–127.

Walmsley, A. 2006. Greenways: multiplying and diversifying in the 21st century. *Landscape and Urban Planning* 76: 252–290.

Wang, S., and B. Fu. 2013. Trade-offs between forest ecosystem services. *Forest Policy and Economics* 26: 145–146.

Weber, T., A. loan, and J. Wolf. 2006. Maryland's Green Infrastructure Assessment: Development of a comprehensive approach to land conservation. *Landscape and Urban Planning* 77: 94–110.

Weber, T.C., and W.L. Allen 2010. Beyond on-site mitigation: An integrated, multi-scale approach to environmental mitigation and stewardship for transportation projects. *Landscape and Urban Planning 96: 240–256.*

Wickham, J.D., K.H. Riitters, T.G. Wade, and P. Vogt. 2010. A national assessment of green infrastructure and change for the conterminous United States using morphological image processing. *Landscape and Urban Planning* 94: 186–195.

Wittmer, H., and H. Gundimeda. 2012. *The economics of ecosystems and biodiversity in local and regional policy and management.* Earthscan, London, New York.

Wolch, J., J. Newell, M. Seymour, H.B. Huang, K. Reynolds, and J. Mapes. 2010. The forgotten and the future: reclaiming back alleys for a sustainable city. *Environment and Planning* 42: 2874–2896.

Wurster, D., and M. Artmann. (2014). Non-monetary assessment of urban ecosystem services on site level – development of a methodology for a standardized selection, mapping and assessment of representative sites. In *AMBIO: A Journal of the Human Environment*: this issue.

Young, R.F. 2011. Planting the Living City Best Practices in Planning Green Infrastructure-Results From Major U.S. Cities. *American Planning Association* 77: 368–381.

Young, R.F., and E.G. McPherson. 2013. Governing metropolitan green infrastructure in the United States. *Landscape and Urban Planning* 109: 67–75.

Yu, D., Y. Jiang, M. Kang, Y. Tian, and J. Duan. 2011. Integrated Urban Land-Use Planning Based on Improving Ecosystem Service: Panyu Case, in a Typical Developed Area of China. *Journal of Urban Planning and Development-ASCE* 137: 448–458.

Zmelik, K., S. Schindler, and T. Wrbka. 2011. The European Green Belt: international collaboration in biodiversity research and nature conservation along the former Iron Curtain. *Innovation-The European Journal of Social Science Research* 24: 273–294.
